# Interaction of annexin A6 with alpha actinin in cardiomyocytes

**DOI:** 10.1186/1471-2121-12-7

**Published:** 2011-01-28

**Authors:** Sumita Mishra, Vivek Chander, Priyam Banerjee, Jae G Oh, Ekaterina Lifirsu, Woo J Park, Do H Kim, Arun Bandyopadhyay

**Affiliations:** 1 Indian Institute of Chemical Biology, 4, Raja S. C. Mullick Road, Kolkata. India; 2 Department of Life Science, Gwangju Institute of Science and Technology, Gwangju, Korea

## Abstract

**Background:**

Annexins are calcium dependent phospholipid binding proteins that are expressed in a wide variety of tissues and implicated in various extra- and intracellular processes. In myocardial tissue, annexins A2, A5 and A6 are particularly abundant, of which the expression levels of annexin A6 has been found to be maximal. Conflicting reports from transgenic mice overexpressing annexin A6 or null mice lacking annexin A6 showed imbalances in intracellular calcium turnover and disturbed cardiac contractility. However, few studies have focussed on the signalling module of annexin A6 in the heart either in normal or in pathological state.

**Results:**

To identify the putative binding partners of annexin A6 in the heart, ventricular extracts were subjected to glutathione S-transferase (GST)- annexin A6 pull down assay and the GST- annexin A6 bound proteins were identified by mass spectrometry. The pull down fractions of ventricular extracts with GST-full length annexin A6 as well as GST-C terminus deleted annexin A6 when immunoblotted with anti sarcomeric alpha (α)-actinin antibody showed the presence of α-actinin in the immunoblot which was absent when GST-N terminus deleted annexin A6 was used for pull down. Overexpression of green fluorescent protein (GFP) tagged full length annexin A6 showed z-line like appearance in cardiomyocytes whereas GFP-N termimus deleted annexin A6 was mostly localized to the nucleus. Overexpression of GFP-C terminus deleted annexin A6 in cardiomyocytes showed aggregate like appearance in the cytoplasm. Double immunofluorescent staining of cardiomyocytes with anti annexin A6 and anti sarcomeric α-actinin antibodies showed perfect co-localization of these two proteins with annexin A6 appearing like a component of sarcomere. Transient knockdown of annexin A6 in cardiomyocytes by shRNA significantly enhances the contractile functions but does not affect the z-band architecture, as revealed by α-actinin immunostaining in shRNA treated cells.

**Conclusions:**

In overall, the present study demonstrated for the first time that annexin A6 physically interacts with sarcomeric α-actinin and alters contractility of cardiomyocytes suggesting that it might play important role in excitation and contraction process.

## Background

The annexins constitute a family of highly conserved proteins that are characterized by their Ca^2+^-dependent binding to phospholipids [1]. Annexins are expressed in a wide variety of tissues and implicated in various extra- and intracellular processes including mitogenic signal transduction, differentiation and membrane trafficking events [2]. However, the exact biological role of each annexin remains unknown.

In myocardial tissue, annexins A2, A5 and A6 are particularly abundant [3-7]. AnxA6 is the most abundant annexin in myocardium [8,9]. It is involved in exocytosis, membrane trafficking and Ca^2+ ^signaling [10]. Conflicting reports demonstrated that it is increased at the onset of heart failure in guinea pig [7] and slightly increased or remain unchanged in failing human hearts [11]. Transgenic mice overexpressing AnxA6 developed dilated cardiomyopathy [12], impaired cardiac contractility and showed enhanced intracellular Ca^2+ ^turnover [13]. In contrast, AnxA6 null mice displayed increased rate of Ca^2+ ^removal in myocytes and enhanced contractility [14].

Annexins are exemplified by a bipartite organization of a unique N terminal domain and C terminal core domain that varies in length and amino acid composition. The N terminal region is thought to confer functional diversity to the annexin protein. The C terminal domain is formed by either a four or eightfold (in case of AnxA6) repeats of approximately 70 amino acid, each repeat carrying a Ca^2+ ^binding site [15].

The mechanism by which AnxA6 alters contractile functions at the cellular level is not clear. We hypothesized that AnxA6 might physically interact with the sarcomeric proteins in cardiomyocytes to alter the contractile functions of heart. Therefore, to gain insight into the functional role of AnxA6, we have analysed its interacting partners by mass spectrometry and examined the functional significance of AnxA6 knockdown in cardiomyocytes.

## Results

### Binding partners of AnxA6 in heart

**Figure 1:**
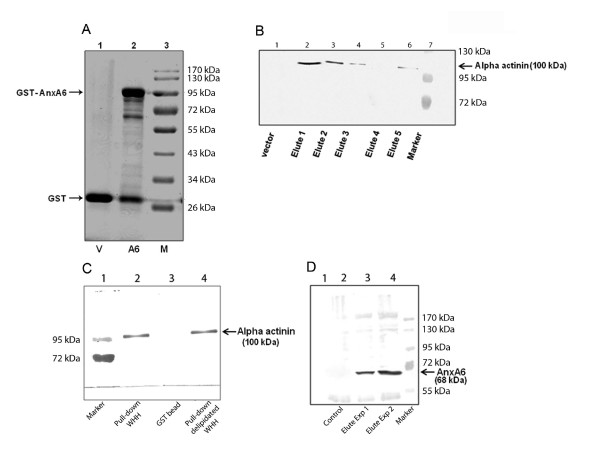
**Identification of putative AnxA6 binding partner(s) in rat heart**. **A**. Purified GST-AnxA6 (A6) affinity beads or GST affinity beads (V) were incubated with NP-40 solubilised WHH (1.5 mg) and the bound proteins (interactome) were separated in 12% SDS-PAGE, stained with Coomassie (lane 2) and analysed via LC MS/MS. **B**. In vitro binding assay: Solubilised whole heart homogenates (WHH) were incubated with GST affinity beads (vector) or AnxA6-GST affinity beads and the bound proteins collected in elute were separated by 10% SDS-PAGE followed by immunoblotting analysis with anti sarcomeric α-actinin antibody. **C**. In vitro binding assay with lipid depleted WHH: Solubilised WHH or delipidated WHH incubated either with GST-AnxA6 affinity beads or GST (vector) affinity beads and the bound proteins in elute were separated by 10% SDS-PAGE followed by immunoblotting analysis with anti sarcomeric α-actinin antibody. **D**. Immunoprecipitation: The whole heart homogenate (300 μg) was subjected to immunoprecipitation with anti sarcomeric α-actinin antibody followed by western blotting with anti-AnxA6 antibody.

To identify the potential interacting proteins of AnxA6 in heart, in vitro binding of whole heart homogenate (WHH) proteins with GST-AnxA6 fusion protein was conducted (Figure 1). The solubilised WHH was applied to GST-AnxA6 bound glutathione-sepharose 4B beads. The proteins not retained by GST-AnxA6 were mostly removed during washing and those bound to GST-AnxA6 were eluted (Figure 1A, lane 2) and subjected to mass spectrometric analysis. The mass spectrometry analysis showed that α-actinin was one of the major proteins bound to GST-AnxA6 (Additional file 1). Therefore, it is apparent that α-actinin might be a potential interacting partner of AnxA6 in the heart. However, the other proteins obtained by mass spectrometry were also likely to interact with AnxA6 since those too were retained by GST-AnxA6 (Additional file 1).

To validate the interaction of AnxA6 with α-actinin in cardiomyocytes, immunoblotting analysis of GST-AnxA6 pull-down fraction with anti sarcomeric α-actinin antibody was conducted. As shown in Figure 1(B), the GST-AnxA6 pull-down fraction of the WHH showed the presence of α-actinin. Since AnxA6 is a phospholipid binding protein and α-actinin is known to bind with phosphatidylinositol bisphosphate (PIP2) [1,16], we examined whether binding of AnxA6 with α-actinin in heart homogenate depends on PIP2. In vitro binding assay clearly demonstrates that delipidation of heart homogenate does not influence interaction of AnxA6 with α-actinin (Figure 1C).

**Figure 2:**
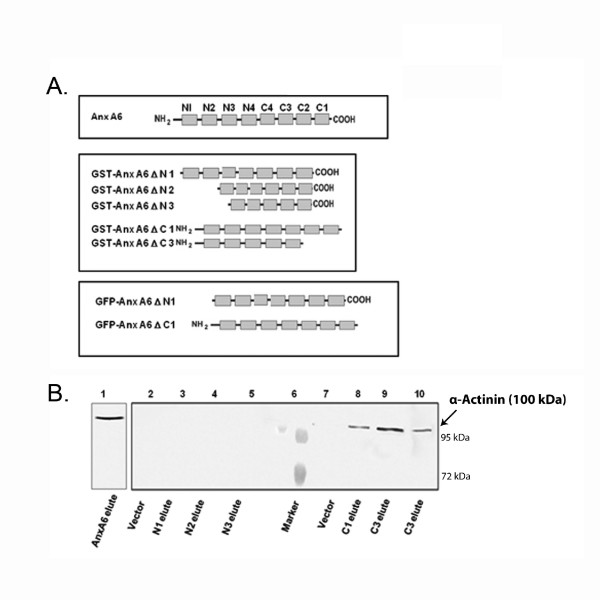
**N-terminus of AnxA6 mediates binding with α-actinin**. **A**. Scheme representing AnxA6 deletion mutants. The full length AnxA6 contains eight repeat domains and associated linker residues (residues 1-673). Deletion mutants GST-AnxA6 ΔN1 lacks N terminal tail and repeat domain 1 (residues 1-89); GST-AnxA6 ΔN2 lacks N terminal tail, repeat domains N1 and N2 (residues 1-163); GST-AnxA6 ΔN3 lacks N terminal tail, repeat domains N1, N2 and N3 (residues 1-250); GST-AnxA6 ΔC1 lacks C terminal tail and repeat domain C1 (residues 600-673); GST-AnxA6 ΔC3 lacks C terminal tail and repeat domains C1, C2 and C3 (residues 435-673). GFP-AnxA6 D N1 lacks N terminal tail and repeat domain 1 (residues 1-89) and GFP-AnxA6 D C1 lacks C terminal tail and repeat domain C1 (residues 600-673). **B**. Solubilised WHH was incubated with GST affinity beads (vector) or various mutants of AnxA6 lacking domains N1-N3 (GST-AnxA6 ΔN1- GST-AnxA6 ΔN3) or mutants lacking domains C1-C3 (GST-AnxA6 ΔC1 and GST-AnxA6 ΔC3). The presence of α-actinin in the bound fractions was detected by immunoblotting analysis with anti sarcomeric α-actinin antibody (arrow head).

To further validate the interaction of AnxA6 with α-actinin, WHH was directly subjected to immunoprecipitation with anti sarcomeric α-actinin antibody followed by western blotting with AnxA6 antibody. As shown in Figure 1(D), AnxA6 was present in the immunoprecipitate indicating that these two proteins interact in vivo. To search for the domains of AnxA6 that could serve for the interaction with α-actinin, in vitro binding assay was conducted using various deletion mutants of GST-AnxA6 fusion proteins (Figure 2B). As shown in Figure 2B, SDS-PAGE analysis followed by in vitro binding experiment with GST fused proteins exhibited significant binding to α-actinin with AnxA6 (lane 1) or mutants lacking domains C1-C3 (GST-AnxA6 ΔC1 - GST-AnxA6 ΔC3; lanes 8-10). However, the interaction between AnxA6 and α-actinin was completely abolished in the AnxA6 mutants lacking domains N1-N3 (GST-AnxA6 ΔN1- GST-AnxA6 ΔN3; lanes 3-5, Figure 2B).

### Localization of GFP-AnxA6 and its deletion mutants in cardiomyocytes

**Figure 3:**
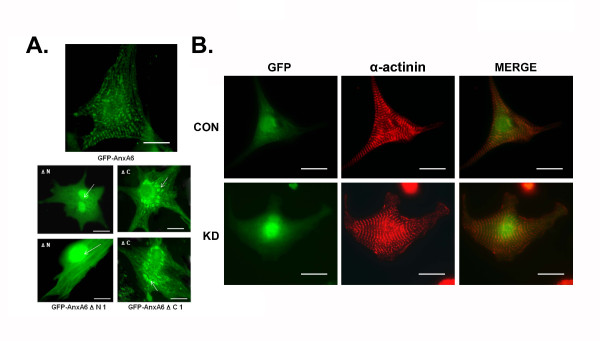
**Subcellular localization of GFP-AnxA6 fusion protein in cardiomyocytes**. **A**. Representative images showing expressions of AnxA6 and its mutants in NRVM. The GFP expression was examined under Olympus (IX71) microscope after 96 hrs of transfection. The GFP-AnxA6 appears to be localized to sarcomeric z-lines (arrow), GFP-AnxA6 ΔN1 is localized to the nucleus (arrow) and GFP-AnxA6 ΔC1 is expressed as aggregate like structures in cytoplasm (arrow), The experiment was repeated with three different batches of myocytes preparation (n = 3). Scale bar represents 10 μm (63×). **B**. Sarcomeric z-line organization showing striations in AnxA6 knocked down cardiomyocytes. NRVM were transfected with AnxA6 shRNA or scrambled constructs and z-lines were visualized by immunofluorescence with α- actinin antibody after 72 h. Transfected cardiomyocytes were selected based on the GFP expression. Representative images were randomly chosen from a pool of cells drawn from 3 independent experiments. Scale bar represents 15 μm (40×).

To examine the subcellular localization of AnxA6 in cardiomyocytes, GFP-AnxA6 was introduced into neonatal rat ventricular myocytes (NRVM) and visualized under fluorescence microscope after 48 hrs. As shown in Figure 3A, GFP-AnxA6 was localized to the cytoskeletal structure, which closely resembles the Z discs typically seen in cardiomyocytes. However, GFP-AnxA6 ΔN1 was mostly localized to the nucleus whereas GFP-AnxA6 ΔC1 was localized to the cytosol and appeared to be large aggregates or vesicles.

To examine whether knock down of AnxA6 expression alters z-band organization, NRVM were transfected with AnxA6 shRNA (KD) or scrambled sequence (CON) and after 72 h subjected to immunofluorescence with anti-sarcomeric α-actinin antibody. As shown in Figure 3B, the striated pattern in cardiomyocytes remained unaffected when AnxA6 was knocked down.

### Co-localization of AnxA6 with α-actinin in cardiomyocytes

**Figure 4:**
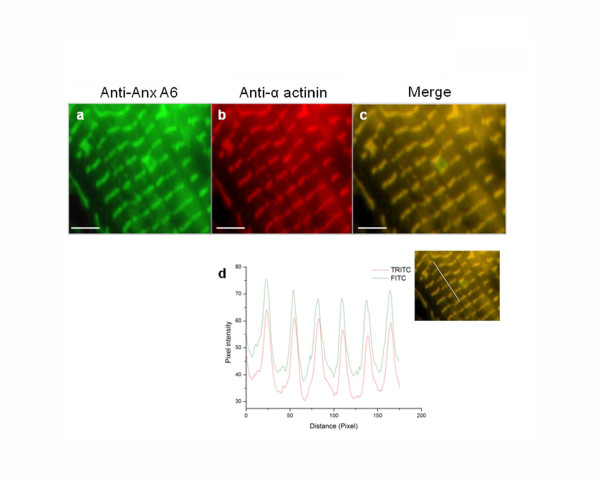
**Colocalization of AnxA6 with a-actinin in cardiomyocytes**. The representative images shown are endogenous expression of AnxA6 (a) and α-actinin (b) in the same cell and their colocalization pattern (c). The line profile for quantification of colocalization (d) was done using the merged image (inset) of AnxA6 and sarcomeric α-actinin. The images represent results of three different experiments with separate batches of myocyte preparation. Scale bar represents 10 μm (100×).

The interaction of AnxA6 with α-actinin *in situ *was examined in NRVM by immunofluorescence microscopy. The pattern of AnxA6 localization in cardiomyocytes was similar to α-actinin and both displayed striated distribution similar to z-discs of sarcomere (Figure 4). The extent of co-localization was enumerated by generating the line profiles of fluorescence intensities of tetramethylrhodamine isothiocyanate (TRITC) for α-actinin and fluorescein isothiocyanate (FITC) for AnxA6 (Figure 4 lower panel). These fluorescence profiles demonstrates (through randomly chosen line over the merged images) the parallel pattern of spatial distribution of AnxA6 and α-actinin signals, indicating a high degree of co-localization of these two proteins, which was further strengthened by Pearson's correlation coefficient as high as 0.898 ± 0.014, obtained from co-localization analysis.

### Involvement of AnxA6 in cardiomyocyte contractility

**Figure 5:**
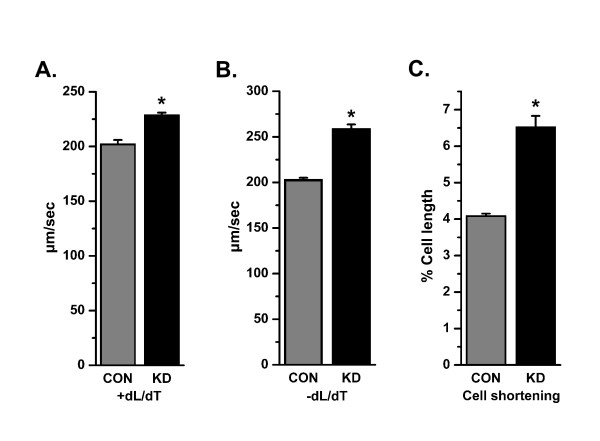
**Contractility in AnxA6 knocked down cardiomyocytes**. ARVM were transfected with AnxA6 shRNA (KD) or scrambled shRNA (CON) constructs and their contractile parameters were determined in GFP-positive cardiomyocytes. Peak cell shortening, percentage of shortened cell length (A); -dL/dt, maximal velocities of cell shortening (B); +dL/dt, maximal velocities of cell relengthening (C) were the contractile parameters. Bars represent the mean ± SEM [n = 6], *p *< 0.05.

To consider the mechanistic effect of AnxA6 in cardiomyocytes, adult rat ventricular myocytes were (ARVM) transfected with AnxA6 shRNA (KD) or scrambled sequence (CON). Mechanical properties were studied in GFP-positive cardiomyocytes using video-based edge detection system (IonOptix) as described in the "Methods" section. AnxA6 shRNA transfected cells exhibited a significant increase in percent shortening (Figure 5C), rate of contraction (+ dL/dt) (Figure 5A) and rate of relaxation (-dL/dt) (Figure 5B) as compared to control cells.

## Discussion

The present study demonstrates AnxA6 to be a member of the z-disc and its involvement in cardiomyocyte contractility. Therefore, altered level of AnxA6 might impair cardiac excitation and contraction cycles, which could explain the contractile abnormality in AnxA6 transgenic as well as knockout mice [12,14]. Though the histochemical localization of Anx6 has been reported in guinea pig heart [7], the present study clearly demonstrates that AnxA6 physically interacts with α-actinin. It is known that α-actinin helps in crosslinking of actin in cardiomyocytes and is an important component of the contractile unit i.e. sarcomere [17].

Since AnxA6 mutant lacking residues 1-250 amino acids (aa) (N3) fails to bind with α-actinin in WHH, it is likely that this region of AnxA6 plays a critical role in mediating binding with α-actinin. On the other hand, residues 435-673 aa, which include domains C1-C3 and C terminal tail, are not involved in this interaction; since the deletion of these residues does not have any effect on in vitro binding. It has been demonstrated earlier that AnxA6 acts as the binding partner of calspectin and regulates bundling of F-actin in vitro [18]. Recently, it has also been shown that AnxA6 interacts with actin exclusively through its N terminal domain in cardiomyocytes [19]. It has been demonstrated earlier that AnxA6 is predominantly located at the sarcolemma and intercalated discs as revealed by the transverse sections of the left ventricle [9,11]. However, the exact subcellular localization of AnxA6 in isolated myocytes was not known. The present study clearly demonstrates the localization of AnxA6 in the z-disc of cardiomyocytes. Furthermore, we specifically show that AnxA6 interacts with α-actinin in vitro as well as in vivo, through its N terminus. Therefore, it is likely that binding of AnxA6 to cardiac sarcomere is mediated via its N terminal domains through α-actinin. Interestingly, z-band organization in cardiomyocytes is not altered when AnxA6 is knocked down. Therefore, absence of AnxA6 does not alter the structural organization of the sarcomeric z-lines.

Interaction of AnxA6 ΔC1 with α-actinin shown by *in vitro *binding experiment indicates that residues required for binding with α-actinin is retained in the purified mutant. Though, *in vitro *binding experiment shows interaction of GST-AnxA6 ΔC1 with α-actinin, it is not clear why the mutant fails to localize to z-discs *in situ*. Such discrepancy appears to be restricted to *in vitro/in vivo *situations solely. Yet, it is plausible that deletion of C terminal sequences affects translocation process of AnxA6 into the z-disc. Moreover, *in situ *over-expression of this mutant most likely induces irregular accumulation of juxtanuclear vesicles of the mutant protein.

It is evident that interaction of AnxA6 with α-actinin occurs via N terminal domain and hence GFP-AnxA6 ΔN1 fails to localize to z-disc when overexpressed in cardiomyocytes. However, it is not clear why this mutant is localized to nucleus. Nuclear localization of AnxA6 in the endothelial and smooth muscle cells of cardiac origin has been reported earlier [8]. In the present study localization of GFP-AnxA6 ΔN1 to the nucleus may be due to the structural transition in the mutant protein regulating its transport and import into the nucleus.

The cytoskeleton of the heart muscle cell is crucially important for the contractile function of the heart. The basic contractile unit of cardiomyocyte is the sarcomere. Actin filaments are directly connected to the z-disc of the sarcomere. α-Actinin is an important actin cross-linking molecule, which exists as an anti-parallel homodimer and orients the actin-binding domains at opposite ends, allowing each dimer to bind two actin filaments, leading to the formation of bundles [17]. The cytoplasmic Ca^2+ ^binds to troponin C, moving the tropomyosin complex off the actin-binding site allowing the myosin head to bind to the actin filament during cardiac excitation and contraction. The intracellular connections between the z-disc of the sarcomere and the sacolemma allow transmission of force developed by the myofilaments [17]. Here we clearly demonstrate the localization of AnxA6 in the z-disc of sarcomere in cardiomyocytes via the mediation of α-actinin.

*In vivo *experiments reported impaired cardiac contractility in AnxA6 transgenic mice whereas enhanced contractility was observed in cardiomyocytes isolated from AnxA6 knock out mice (13, 14). Consistent with these in vivo observations here we show that transient knock down of AnxA6 significantly (P < 0.05) augments percent shortening, rate of shortening (+dL/dT) and rate of relengthening (-dL/dT) as compared to control cardiomyocytes indicating that contractility of cardiomyocytes is enhanced. Hence, AnxA6 likely serves as a regulator of cardiac contractility by its association with the contractile proteins in cardiomyocyte. Therefore, interaction of AnxA6 with α-actinin signifies its potential role in cardiac mechano-transduction.

## Conclusions

This study is a maiden report showing the spatial co-localization and physical interactions of AnxA6 with sarcomeric α-actinin. The functional significance of such interaction might be important for regulating contractile properties of cardiomyocytes. Thus, AnxA6 may have a significant role in cardiac pathophysiology.

## Methods

### Material

Collagenase type 2, anti sarcomeric α-actinin primary antibody, Protein A agarose were purchased from Sigma Chemical Co (St. Louis, MO, USA). All other primary and secondary antibodies were purchased from Santa Cruz Biotechnology (Santa Cruz, USA).

### Animal

Sprague-Dawley male rats for the present study were available from Institute's animal facility. The animals were handled as per the guidelines of the animal ethics committee of this institute and the Committee for the Purpose of Control and Supervision of Experiments on Animals, Ministry of Social Justice and Empowerment, Government of India.

### Preparation of heart homogenates for in vitro binding assay

Cardiac tissue from wild type SD rat was homogenized in an extraction buffer containing 10 mM Tris, pH 8.5, 0.01% (v/v) SDS, 20 mM NaCl, 0.01% (v/v) Nonidet P-40, 5 mM EDTA, 2 mM DTT, and protease inhibitors. The homogenate was centrifuged for 10 min 3000 × g at 4°C, and the supernatant was used for GST pull down assay.

### Generation of GST fusion proteins of AnxA6 and its mutants for in vitro binding assay

cDNA encoding the full-length AnxA6 and its mutants lacking various domains in the amino-terminal and carboxyl terminal regions were cloned into GST fusion vector pGEX-4T-1 (GE-Amersham). Standard molecular cloning techniques were used to manipulate DNA. The primers are listed in additional file 2. The scheme of constructs is summarized in Figure 2A. Expression and purification of the fusion proteins were performed according to the protocol of the manufacturer (GE-Amersham).

### In vitro binding assay

In vitro binding experiment was performed as described earlier [20]. Equal amounts of recombinant GST and GST-AnxA6 were bound to glutathione-sepharose and mixed with 1.5 mg of whole heart homogenates at 4°C for 5 hours. Nonspecific binding was determined by carrying out the binding with GST, instead of GST-AnxA6. The Co-complexed proteins after washing in the cold washing buffer (10 mM Tris, pH 8.5, 0.01% (v/v) Nonidet P-40, 5 mM EDTA, 2 mM DTT, 0.05% (v/v) SDS) were eluted with 1 volume of 20 mM glutathione and 150 mM NaCl in 50 mM Tris/HCl, pH 8. After washing the proteins were separated by heating for 5 mins at 90°C in the SDS-PAGE sample buffer (50 mM Tris, pH 6.8, 50 mM DTT, 2% (v/v) SDS, 0.2% (v/v) bromphenol blue, 10% (v/v) glycerol) and were then detected by the Coomassie blue staining. Interacting proteins were analysed using the LC MS or subjected to immunoblotting analysis for validation.

### Mass spectrometric analysis to search for the interacting partners

Interacting proteome were analysed using LC MS/MS as described elsewhere [21]. Raw files were searched for fully tryptic peptides against the IPI Rat database using Sequest software.

### Western blot analysis

Equal amounts of GST or GST-AnxA6 pull down fractions were resolved onto 10% SDS polyacrylamide gels and subjected to immunoblotting analysis with α-actinin antibody following previously described method [22].

### Delipidation of WHH

Delipidation of WHH was done with little modification of previously described method. [23,16].

### Immunoprecipitation

For immunoprecipitation (IP) experiment, α-actinin antibody was pre-incubated with Protein A agarose for 4 hours at 4°C. After the protein A agarose got saturated with the antibody, it was added to 1 mg of WHH and was incubated overnight at 4°C. The IP pellets thus obtained were washed twice for 15 minutes each using cold wash buffer and one wash using PBS-T at 4°C. Identification of AnxA6 in immunoprecipitate was performed by western blot analysis with anti-AnxA6 antibody.

### Isolation rat ventricular cardiomyocytes

Neonatal rat ventricular cardiomyocytes (NRVM) were prepared by previously described method [24]. Adult rat ventricular cardiomyocytes (ARVM) from Sprague-Dawley rats were isolated as reported earlier with some minor modifications [25]. The pellets contained 90-97% rod shaped viable ventricular myocytes, were resuspended in glucose-free KH buffer. Approximately 5.0 × 10^4 ^rod-shaped cells were plated on 18-mm laminin-coated coverslips and maintained in a 5% CO_2 _incubator.

### Cloning for over-expression of AnxA6 into cardiomyocytes

cDNAs encoding full length AnxA6 or AnxA6 lacking residues 1-89 aa (GFP-AnxA6 ΔN1) or AnxA6 lacking residues 600-673 aa (GFP-AnxA6 ΔC1) were amplified using gene specific primers and cloned into HindIII/PstI sites of pEGFP-N1 vector (BD Biosciences Clontech). About 2.8 μg of DNA was used to transfect freshly isolated NRVM (8 × 10^5 ^cells) through electroporation using 'Rat Cardiomyocyte-Neonatal Nucleofector TM Kit (amaxa GmbH)', following manufacturer's protocol [26]. Expression of EGFP-AnxA6 and its mutants were monitored at 488 nm. The empty vector was also transfected as control.

### Annexin A6 knock down

To transiently knock down AnxA6 expression in cardiomyocytes, 29 mer shRNA construct against rat AnxA6 in pGFP-V-RS vector was obtained (OriGene Technologies Inc, Rockville, MD, USA). Scrambled shRNA was employed as control. Transfection was performed using Lipofectamine™ LTX reagent (Invitrogen, Carlsbad, USA). About 30% ARVM were found to be transfected with shRNA constructs based on GFP signals. Due to the limitation of transfection efficiency in ARVM, knock down efficiency was quantified in human embryonic kidney (HEK) cells where nearly 100% cells were GFP positive [Additional file 3A]. Cell extracts were prepared 72 h after transfection with shRNA and immunoblotting with anti-AnxA6 antibody was conducted. As shown in additional file 3B and 3C, 60% knock down of Anx A6 was achieved with AnxA6 shRNA sequence in comparison to the scrambled shRNA sequence in the transfected cells.

### Cell contractility measurement

The mechanical properties of ARVM were assessed using a video-based edge detection system (IonOptix) as previously described [26]. In brief, laminin-coated coverslips with cells attached were placed in a chamber mounted on the stage of an inverted microscope (Nikon Eclipse TE-100F) and superfused (about 1 ml/min at 25°C) with Tyrode buffer (137 mM NaCl, 5.4 mM KCl, 1 mM CaCl2, 1 mM MgCl2, 10 mM glucose, and 10 mM HEPES (pH 7.4)). The cells were field stimulated at a frequency of 1 Hz (30 V) using a STIM-AT stimulator/thermostat placed on HLD-CS culture chamber/stim holder (Cell Micro Controls). Changes in cell length during shortening and relengthening were captured in GFP-positive cardiomyocytes and analyzed using soft edge software (IonOptix).

### Immunofluorescence

The localization of AnxA6 and α-actinin were examined by immunofluorescence using specific antibodies against each protein as described earlier [24].

### Statistical analysis

The results are presented as the mean ± S.E.M. Each experiment was conducted independently at least three times. A level of p < 0.05 was considered the threshold for statistical significance.

## Competing interests

The authors declare that they have no competing interests.

## Authors' contributions

SM was responsible for in vitro binding assay, mass spec. analysis, cell culture, immunofluorescence, generation of mutants, immunoprecipitation, contractility measurement and manuscript preparation. VC was responsible for dilipidation, western blotting and immunofluorescence experiments. PB validated shRNA constructs and helped in manuscript preparation. EL and GJP isolated myocytes and conducted contractility experiments. All authors made substantial contributions to the design of the experiments, acquisition and analysis of data and preparation of the figures. WJP supervised contractility studies. DHK supervised part of the studies. AB supervised these studies and prepared the manuscript. All authors read and approved the final manuscript.

## Supplementary Material

Additional file 1 **List of the possible interacting partners of AnxA6**. Click here for file

Additional file 2 **List of primers**. Click here for file

Additional file 3 **A. Expression of pGFP-V-RS/AnxA6 shRNA construct in HEK cells**. Transfected cells were analyzed after 72 hours for GFP expression and approx.100% cells were found to be GFP-positive. Scrambled showed similar results. Scale bar represents 100 μm (10×). **B**. Immunoblot analysis were performed in AnxA6 shRNA or scrambled transfected HEK cells at 72 hours post transfection with anti AnxA6 antibody or anti actin antibody (Loading control). **C**. Quantification of AnxA6 knockdown in HEK cells. Densitometry analysis (NIH ImageJ) shows approx. 60% knockdown of AnxA6 as compared to scrambled. Data were normalized to the values of loading control and plotted as mean ± S.E.M. *p *< 0.05. Click here for file

## References

[B1] Rescher U Gerke V Annexins-unique membrane binding proteins with diverse functions J Cell Sci 2004 117 2631 2639 10.1242/jcs.01245 15169834

[B2] Donato R Russo-Marie F The annexins: structure and functions Cell Calcium 1999 26 85 89 10.1054/ceca.1999.0079 10598271

[B3] Benevolensky D Belikova Y Mohammadzadeh R Trouve P Marotte F Russo-Marie F Samuel JL Charlemagne D Expression and localization of annexins II, V, and VI in myocardium from patients with end-stage heart failure Lab Invest 2000 80 2 123 33 10701682 10.1038/labinvest.3780016

[B4] Camors E Monceau V Charlemagne D Annexins and Ca^2+ ^handling in the heart Cardiovasc Res 2005 65 793 802 15721859 10.1016/j.cardiores.2004.11.010

[B5] Iida H Hatate T Shibata Y Immunocytochemical localization of 67 kD Ca^2+^-binding protein (p67) in ventricular, skeletal, and smooth muscle cells J Histochem Cytochem 1992 40 1899 1907 1453007 10.1177/40.12.1453007

[B6] Jans SWS van Bilsen M Reutelingsperger CPM Borgers M de Jong YF van der Vusse GJ Annexin V in the adult rat heart: Isolation, location and quantitation J Mol Cell Cardiol 1995 27 335 348 7760355 10.1016/s0022-2828(08)80031-4

[B7] Trouve P Legot S Belikova I Marotte F Benevolensky D Russo-Marie F Samuel JL Charlemagne D Localization and quantification of cardiac annexins II, V and VI in hypertensive guinea pigs Am J Physiol 1999 276 H1159 H1166 10199838 10.1152/ajpheart.1999.276.4.H1159

[B8] Doubell AF Lazure C Charbonneau C Thibault G Identification and immunolocalization of annexins V and VI, the major cardiac annexins, in rat heart Cardiovasc Res 1993 27 1359 1367 8252600 10.1093/cvr/27.7.1359

[B9] Luckcuck T Trotter PJ Walker JH Localization of annexin VI in the adult and neonatal heart Cell Biol Int 1998 22 199 205 9974214 10.1006/cbir.1998.0244

[B10] Grewal T Heeren J Mewawala D Schnitgerhans T Wendt D Salomon G Enrich C Beisiegel U Jäckle S Annexin VI stimulates endocytosis and is involved in the trafficking of low density lipoprotein to the prelysosomal compartment J Biol Chem 2000 275 33806 33813 10940299 10.1074/jbc.M002662200

[B11] Matteo RG Moravec CS Immunolocalization of annexins IV, V and VI in the failing and non-failing human heart Cardiovasc Res 2000 45 961 970 10728422 10.1016/s0008-6363(99)00409-5

[B12] Gunteski-Hamblin AM Song G Walsh RA Frenzke M Boivin GP Dorn GW Kaetzel MA Horseman ND Dedman JR Annexin VI overexpression targeted to heart alters cardiomyocyte function in transgenic mice Am J Physiol 1996 270 H1091 H1100 8780208 10.1152/ajpheart.1996.270.3.H1091

[B13] Song G Campos B Wagoner LE Dedman JR Walsh RA Altered cardiac annexin mRNA and protein levels in the left ventricle of patients with end -stage heart failure J Mol Cell Cardiol 1998 30 443 451 9515022 10.1006/jmcc.1997.0608

[B14] Song G Harding SE Duchen MR Tunwell R Gara PO Moss SE Altered mechanical properties and intracellular calcium signaling in cardiomyocytes from annexin 6 null-mutant mice FASEB J 2002 16 622 624 11919174 10.1096/fj.01-0892fje

[B15] Raynal P Pollard HB Annexins: the problem of assessing the biological role for a gene family of multifunctional calcium- and phospholipid-binding proteins Biochem Biophys Acta 1994 1197 63 93 8155692 10.1016/0304-4157(94)90019-1

[B16] Fukami K Furuhashi K Inagaki M Endo T Hatano S Requirement of phosphatidylinositol 4, 5-bisphosphate for actinin function Nature 1992 359 150 152 1326084 10.1038/359150a0

[B17] Frank D Kuhn C Katus HA Frey N The sarcomeric Z-disc: a nodal point in signalling and disease J Mol Med 2006 84 446 468 16416311 10.1007/s00109-005-0033-1

[B18] Watanabe T Inui M Chen BY Iga M Sobue K Annexin VI-binding proteins in brain. Interaction of annexin VI with a membrane skeletal protein, calspectin (brain spectrin or fodrin) J Biol Chem 1994 269 17656 17662 8021276

[B19] Locate S Colyer J Gawler DJ Walker JH Annexin A6 at the cardiac myocyte sarcolemma - evidence for self-association and binding to actin Cell Biol Int 2008 32 1388 1396 18782625 10.1016/j.cellbi.2008.08.009

[B20] Shin DW Ma J Kim DH The asp-rich region at the carboxy terminus of calsequestrin binds to Ca^2+ ^and interacts with triadin FEBS Lett 2000 486 178 182 11113462 10.1016/s0014-5793(00)02246-8

[B21] Luo J Hill BG Gu Y Cai J Srivastava S Bhatnagar A Prabhu SD Mechanisms of acrolein-induced myocardial dysfunction: implications for environmental and endogenous aldehyde exposure Am J Physiol Heart Circ Physiol 2007 293 H3673 H3684 17921335 10.1152/ajpheart.00284.2007

[B22] De K Ghosh G Datta M Konar A Bandyopadhyay J Bandyopadhyay D Bhattacharya S Bandyopadhyay A Analysis of differentially expressed genes in hyperthyroid-induced hypertrophied heart by cDNA microarray J Endocrinol 2004 182 303 314 10.1677/joe.0.1820303 15283691

[B23] Schröder B Hasilik A A protocol for combined delipidation and subfractionation of membrane proteins using organic solvents Analytical Biochemistry 2006 357 144 146 16919231 10.1016/j.ab.2006.05.035

[B24] Piper HM Schwartz P Hutter JF Spieckermann PG Energy metabolism and enzyme release of cultured rat heart muscle cells during anoxia J Mol Cell Cardiol 1984 16 995 1007 6394766 10.1016/s0022-2828(84)80013-9

[B25] Jeong D Kim JM Cha H Oh JG Park J Yun SH Ju ES Jeon ES Hajjar RJ Park WJ PICOT attenuates cardiac hypertrophy by disrupting calcineurin-NFAT signalling Circ Res 2008 102 711 719 18258855 10.1161/CIRCRESAHA.107.165985

[B26] Cha H Kim JM Oh JG Jeong MH Park CS Park J Jeong HJ Park BK Lee YH Jeong D Yang DK Bernecker OY Kim do H Hajjar RJ Park WJ PICOT is a critical regulator of cardiac hypertrophy and cardiomyocyte contractility J Mol Cell Cardiol 2008 6 796 803 10.1016/j.yjmcc.2008.09.124 PMC2752880 18929570

