# Supplementary material for: Interaction of annexin A6 with alpha actinin in cardiomyocytes
Source: BMC Cell Biol. 2011 Jan 28;12:7. doi: 10.1186/1471-2121-12-7 (PMC3037912; doi:10.1186/1471-2121-12-7)
Supplement: Additional file 1 — List of the possible interacting partners of AnxA6. [file 1471-2121-12-7-S1.DOC]

**List of the possible interacting partners of AnxA6:**

| Sl No. | Name of the protein/peptide |
| --- | --- |
| 1  2  3  4  5  6  7  8  9  10  11  12  13  14  15  16  17  18  19  20  21 | Siahbp1 RNA-binding protein SiahBP  Nup93 Nucleoporin 93  **α actinin**  Ftsj3 Putative rRNA methyltransferase 3  Prap1 Poly[ADP-ribose] polymerase 1  Svs1 basic protein  MLL Zinc finger protein HRX  HNRPUL-1  Hnrpm Isoform 1 of Heterogeneous nuclear ribonucleoprotein M  Foxp3 Forkhead box protein P3  Pou4f1 POU domain, class 4, transcription factor 1  Sox14_predicted similar to Transcription factor SOX-14  Hnf1b Isoform 1 of Hepatocyte nuclear factor 1-beta  Tada3l Isoform 1 of Transcriptional adapter 3-like  Sorting nexins  Legumin (endosomal/lysosomal degradation pathway)  Calpain (protease involved in apoptosis)  Contactin  Pcdhb protein (calcium dependent cadherin)  Desmoglein 2(cadherin)  Dynine (microtubule associated protein) |

Protein bands were excised from Coomassie Blue-stained polyacrylamide gels and digested with trypsin, followed by mass spectrometry analysis of the protein fragments.
