# Supplementary material for: Interaction of annexin A6 with alpha actinin in cardiomyocytes
Source: BMC Cell Biol. 2011 Jan 28;12:7. doi: 10.1186/1471-2121-12-7 (PMC3037912; doi:10.1186/1471-2121-12-7)
Supplement: Additional file 2 — List of primers. [file 1471-2121-12-7-S2.DOC]

**List of primers:**

| Clone | Forward Primer | Reverse Primer |
| --- | --- | --- |
| **GST-AnxA6** | GCGCGTCGACTGATGGCCAAAATAGCACAG | GTCGGCGGCCGCTTAGTCTTCTCCACCAC |
| **GST-AnxA6 Δ N 1** | GCGCGTCGACTGATGGGCACTGATGAGAAGTGC | GTCGGCGGCCGCTTAGTCTTCTCCACCAC |
| **GST-AnxA6 Δ C 1** | GCGCGTCGACTGATGGCCAAAATA GCACAG | CGGCGGCCGCCGGCTCCCTCCATAGC |
| **GST-AnxA6 Δ C 3** | GCGCGTCGACTGATGGCCAAAATA GCACAG | CGGCGGCCGCCGGCACCCTTCATGGAC |
| **GST-AnxA6 Δ N 2** | GCGTCGACTGATGGGAACAGATGAGGCCCAG | GTCGGCGGCCGCTTAGTCTTCTCCACCAC |
| **GST-AnxA6 Δ N 3** | CGCGTCGACTGATGGGGACTCGAGACAACACT | GTCGGCGGCCGCTTAGTCTTCTCCACCAC |
| **GFP-AnxA6** | CCCAAGCTTATGGCCAAAATAGCAC | AACTGCAGGTAGTCTTCTCCACCACAG |
| **GFP-AnxA6 Δ C** | CCCAAGCTTATGGCCAAAATAGCAC | GCGCAACTGCAGTGTCTCCACTGGGTGT GTC |
| **GFP-AnxA6 Δ N** | CCCAAGCTTATGGGCACTGATGAGAAGTGC | AACTGCAGGTAGTCTTCTCCACCACAG |
