# Supplementary figures and images for: Interaction of annexin A6 with alpha actinin in cardiomyocytes
Source: BMC Cell Biol. 2011 Jan 28;12:7. doi: 10.1186/1471-2121-12-7 (PMC3037912; doi:10.1186/1471-2121-12-7)

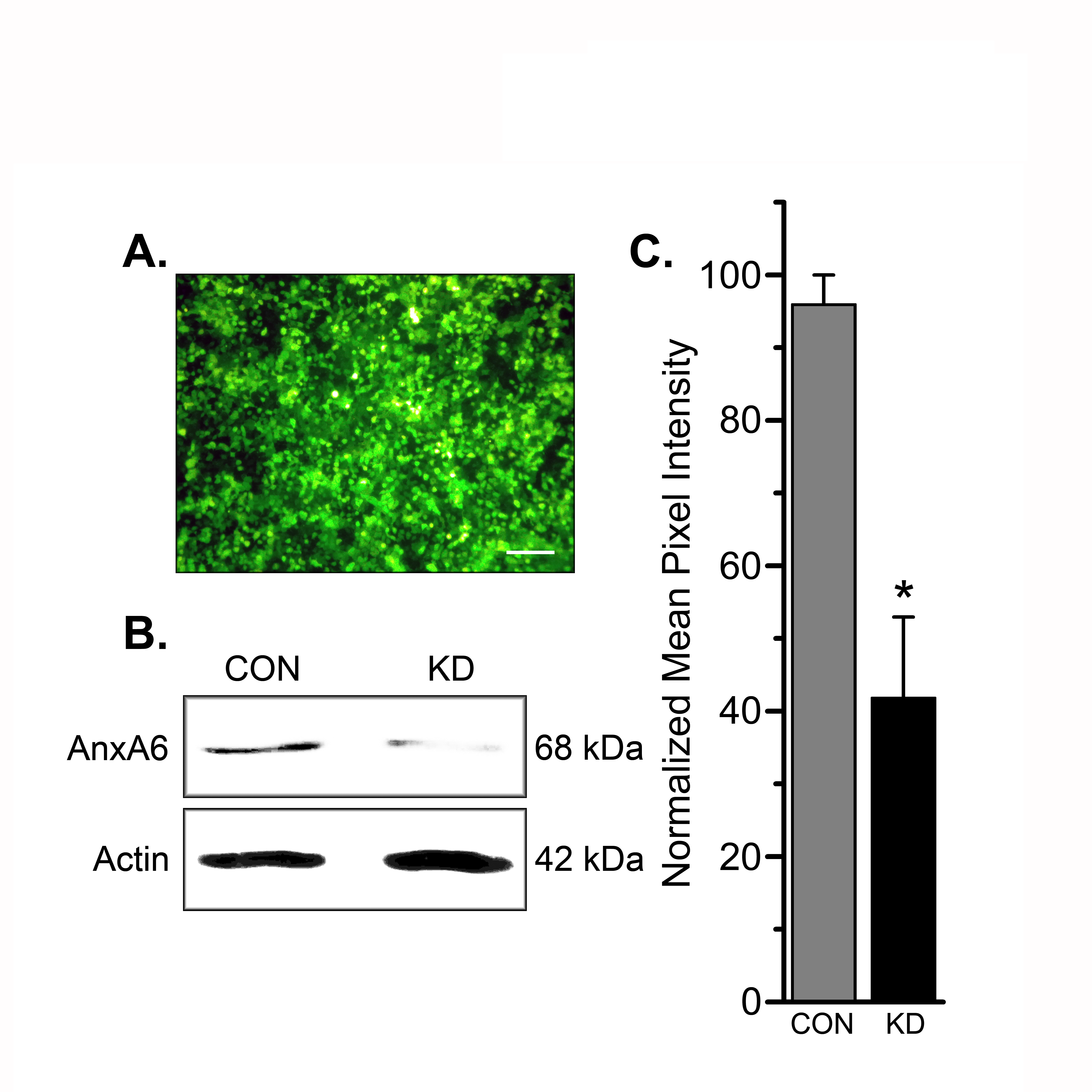

Supplement: Additional file 3 — A. Expression of pGFP-V-RS/AnxA6 shRNA construct in HEK cells. Transfected cells were analyzed after 72 hours for GFP expression and approx.100% cells were found to be GFP-positive. Scrambled showed similar results. Scale bar represents 100 μm (10×). B. Immunoblot analysis were performed in AnxA6 shRNA or scrambled transfected HEK cells at 72 hours post transfection with anti AnxA6 antibody or anti actin antibody (Loading control). C. Quantification of AnxA6 knockdown in HEK cells. Densitometry analysis (NIH ImageJ) shows approx. 60% knockdown of AnxA6 as compared to scrambled. Data were normalized to the values of loading control and plotted as mean ± S.E.M. p < 0.05. [file 1471-2121-12-7-S3.TIFF]
